# A Modified Double-Eyelid Blepharoplasty: Tarsus Linkage Mechanism

**DOI:** 10.1007/s00266-023-03444-6

**Published:** 2023-06-29

**Authors:** Xin Lu, Yunyan Ye, Yuxian Qian

**Affiliations:** 1 Department of Ophthalmology, Ningbo Medical Center Lihuili Hospital, Medical School of Ningbo University, Ningbo, 315000 Zhejiang People’s Republic of China; 2 Ya Su Medical Beauty Clinic, Ningbo, 315000 Zhejiang People’s Republic of China

**Keywords:** Blepharoplasty, Double-eyelid, Selective opening, Technique modification

## Abstract

**Background:**

Double-eyelid blepharoplasty is the most popular plastic surgery in East Asia. The incisional methods are divided into two schools. The traditional method produces a stable eyelid, but will leave a postoperative scar. The other is represented by “Park,” creating dynamic double-eyelid technology. Its advantage is that there is only mild scarring, but its disadvantages are asymmetry, corneal exposure, and loss of the palpebral furrow. Due to these various complications, we here propose an improved incisional blepharoplasty with the tarsus linkage mechanism.

**Methods:**

This work covers 482 patients who underwent surgery from March 2018 to March 2022. All patients completed 6 months of postoperative follow-up. The basic procedure described here involves removing the pre-tarsal tissue without completely incising the orbicularis and suturing the orbicularis and the tarsus into a unit. This connection provides a more robust and stable eyelid adhesion.

**Results:**

As reported by physicians, 412 patients (85.5%) had satisfactory results, 69 patients (14.3%) had somewhat satisfactory results, and 1 patient (0.2%) had unsatisfactory results. As reported by the patients, 424 patients (88.0%) were satisfied, 57 patients (11.8%) were somewhat satisfied, and 1 patient (0.2%) was unsatisfied.

**Conclusion:**

This study proposes a modified double-eyelid blepharoplasty with the tarsus linkage mechanism. It is suitable for most primary eye cases, particularly in patients with lax upper lid skin and high levels of upper orbital fat.

**Level of Evidence V:**

This journal requires that authors assign a level of evidence to each article. For a full description of these Evidence-Based Medicine ratings, please refer to the Table of Contents or the online Instructions to Authors www.springer.com/00266.

**Supplementary Information:**

The online version contains supplementary material available at 10.1007/s00266-023-03444-6.

## Introduction

Because more than half of East Asians naturally lack an upper eyelid crease, often referred to as a “single eyelid,” double-eyelid blepharoplasty has been the most popular cosmetic procedure in East Asia for many years [1].

Depending on the surgical approach, blepharoplasty can be divided into incisional and suture methods, both of which are designed to mimic the physiological levator-skin connection [2, 3]. Scar formation between the levator tendon membrane and the lower flap of the eyelid is the basic fixation mechanism for this type of blepharoplasty.

The incisional methods are divided into two schools. One is the traditional incision method [4], which relies on the tarsus movement to drive the adherent lower flap eyelid skin to form the eyelid line. It is classified as a passive static double-eyelid technique. The advantage of this technique is that it produces a stable eyelid. The disadvantage is that the postoperative scar is readily visible when the eyes are closed. Another technique, the levator dynamic system, is directly or indirectly used to create a dynamic blepharoplasty technique [5–7]. This is represented by Park [8]. The advantage is that it corrects complications associated with inadequate strength of the levator with only minimal long-term postoperative scarring [7, 9]. The most common complications are asymmetry, corneal exposure, and loss of the palpebral furrow loss [9].

Our study proposes a modified idea of an incisional blepharoplasty to compensate for those shortcomings. Thanks to the widespread selection of lumpectomy in surgery, many surgeons have pursued the goal of minimizing disturbance to and disruption of the local anatomy and improving the overall internal environment of the procedure while retaining effectiveness. Our method pioneers the use of selective opening to remove pre-tarsal fatty tissue while preserving the integrity of the orbicularis to the greatest extent possible. Most previous blepharoplasties have involved complete incision of the orbicularis muscle to create a stable crease in this area [10]. However, these methods also disrupt the integrity and circulation of the orbicularis, leading to eyelid scars and prolonged swelling.

This method draws on the Park method to preserve the orbicularis, and it also draws on the traditional method of forming a fixed adhesion between the pre-tarsal skin and the tarsus that prevents the loosening of double-eyelid lines. Our goal being to preserve the integrity of the orbicularis to the greatest extent possible, we removed pre-tarsal fatty tissue to establish a more stable and minimally invasive form of incisional blepharoplasty.

## Patients and Methods

### Clinical Information

Inclusion criteria: 1. age ≥ 18 years old; 2. patients with no ptosis; 3. patients who willing to undergo blepharoptosis. Exclusion criteria: 1. history of blepharoplasty; 2. scar constitution. All patients in this group provided signed informed consent and agreed to the data used for this study.

### Surgical Procedure

The method consists of the following steps.

#### Preoperative Design

According to the preset design requirements, two lines were drawn on the upper eyelid: the double-eyelid line and the skin removal line.

#### Local Anesthetic

The procedure was performed under local anesthesia in conjunction with an adequate local anesthetic administered subcutaneously (2% lidocaine combined with epinephrine 1: 100,000).

#### Skin Removal

The skin was incised until the orbicularis was exposed using a #11 blade according to the design.

#### Selective Opening of the Orbicularis Oculi Muscle

**Fig. 1:**
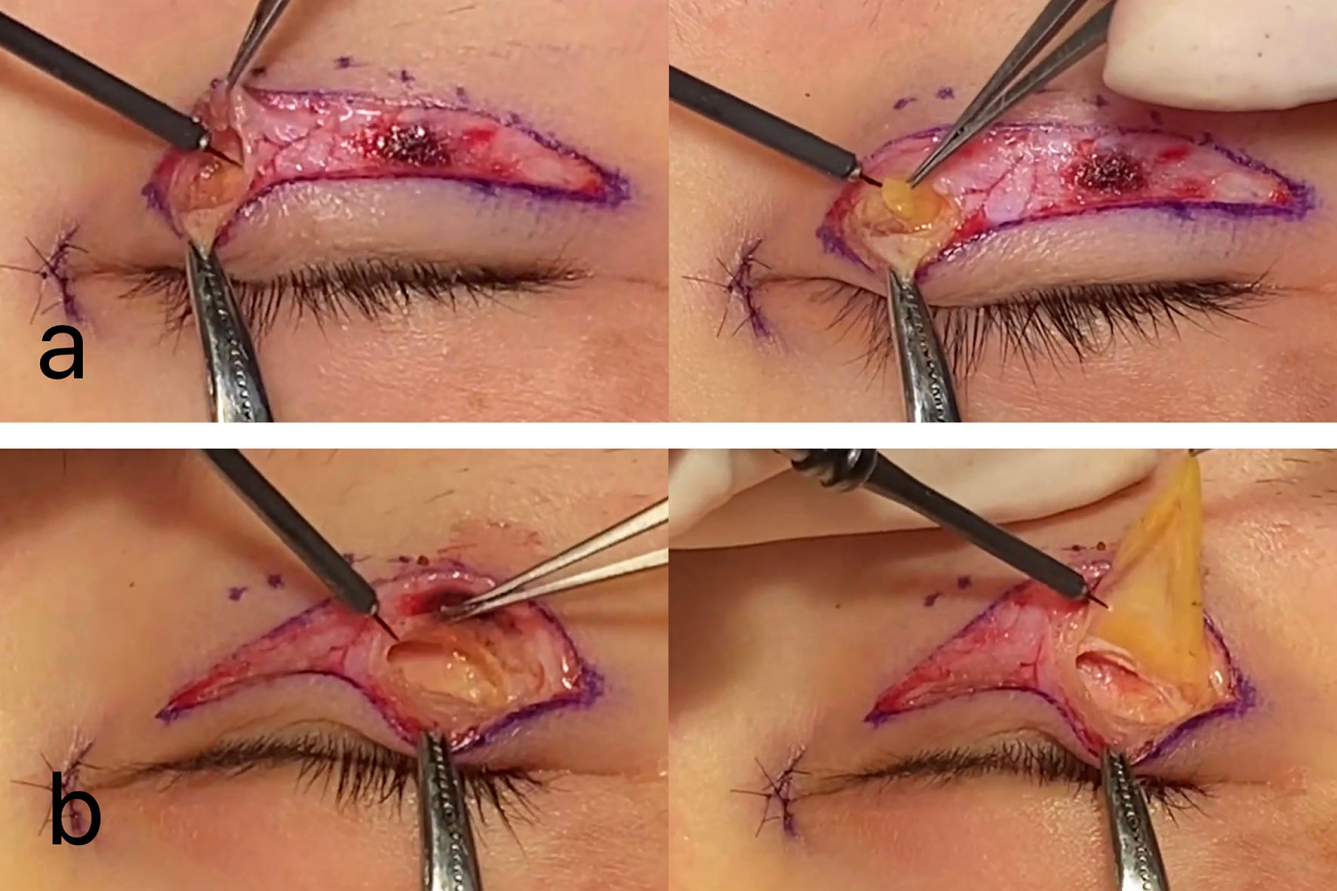
Two openings were selected to remove the pre-tarsal tissue and the herniated orbital fat

To keep the orbicularis intact, two preset points were selected to open to remove the pre-tarsal fascia tissue. These were located directly above the pupil and at the lateral canthal area. The length of the opening at the lateral canthal area was 5 mm from which the pre-tarsal tissue and the herniated orbital fat were removed (Fig. 1).

#### Tarsus Linkage Mechanism

**Fig. 2:**
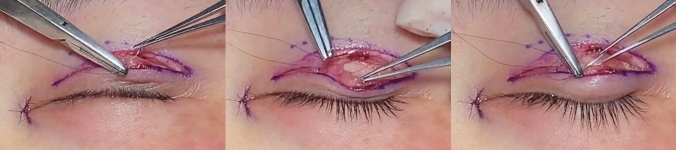
A 7-0 nylon suture was passed through the orbicularis and the tarsus to form the tarsus linkage mechanism

**Fig. 3:**
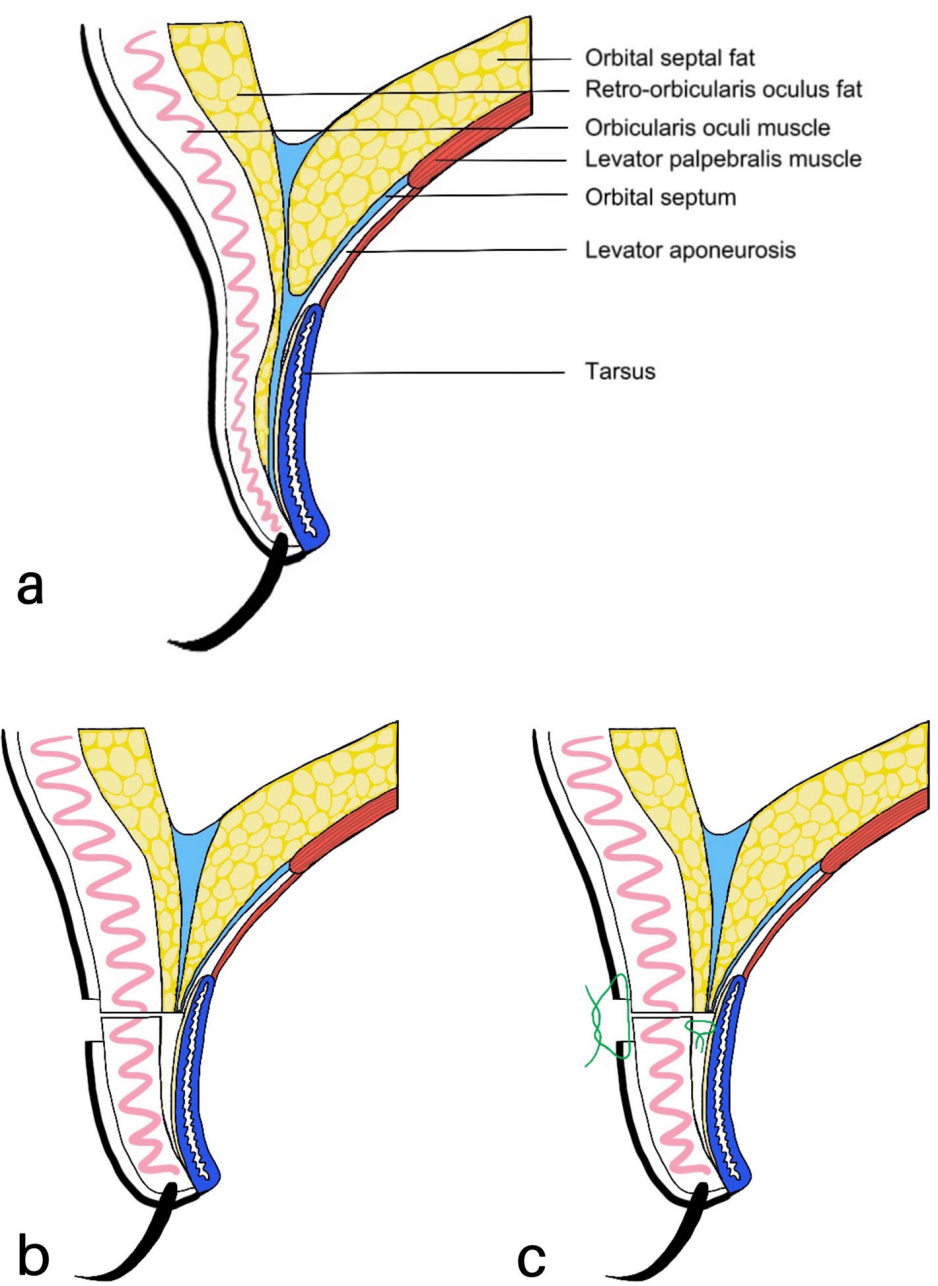
**a** Schematic diagram of the upper eyelid. **b** Scar healing by removing the pre-tarsal tissue created surface adhesion. **c** Suture of orbicularis and tarsus formed a levator aponeurosis–tarsus–orbicularis–skin linkage mechanism

Reinforcement of the fixation was accomplished through orbicularis–tarsus closure with a 7-0 nylon suture in each selective opening (Fig. 2). Removing pre-tarsus tissue and performing a fixation suture made the lower flap into a composite unit. It formed a levator aponeurosis–tarsus–orbicularis–skin linkage mechanism (Fig. 3). The skin incisions were then closed with 7-0 nylon suture in counterpoint.

### Efficacy Evaluation

Follow-up was performed at 1 week, 1 month, and 6 months after surgery. The quality of the blepharoplasty was scored by the two surgeons who had not operated on that specific patient and by the patients themselves (criteria given in Table 1). Scores of 8–10 were considered satisfactory, 5–7 somewhat satisfactory, and 0–4 unsatisfactory.

**Table 1:** Satisfaction criteria for blepharoplasty

	2	1	0
Width	Satisfactory	General	Too wide or too narrow, needs to be adjusted
Eyelid curvature	Smooth and symmetrical	General	Needs to be adjusted
Granuloma	None	Mildly bloated	Needs to be adjusted
Crease dissolved	None	A little lighter	Needs to be adjusted
Incision scar	Flat and unnoticeable	Not very flat	Hypertrophy or depression

## Results

A total of 482 patients (431 women and 51 men) underwent this blepharoplasty from March 2018 to March 2022. The mean age was 27 years (range 18–62). All indicators completed 6 months of postoperative follow-up.

As reported by physicians, 412 patients (85.5%) had satisfactory results, 69 patients (14.3%) had somewhat satisfactory results, and 1 patient (0.2%) had unsatisfactory results. As reported by the patients, 424 patients (88.0%) were satisfied, 57 patients (11.8%) were somewhat satisfied, and 1 patient (0.2%) was unsatisfied.

**Fig. 4:**
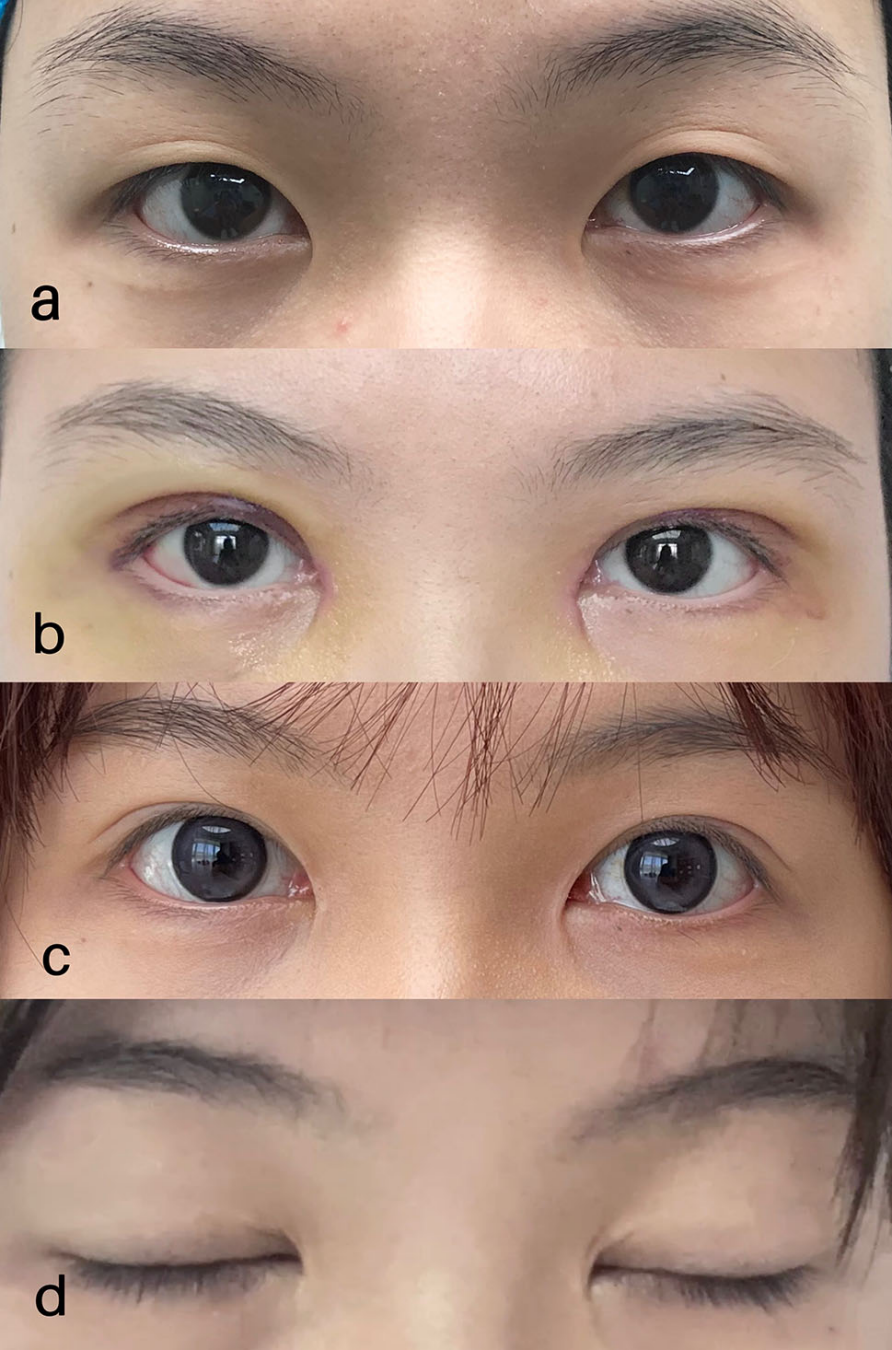
**a** Preoperative view. **b** Seven days after blepharoplasty. **c** Six months after blepharoplasty. **d** Closed eyes after six months

**Fig. 5:**
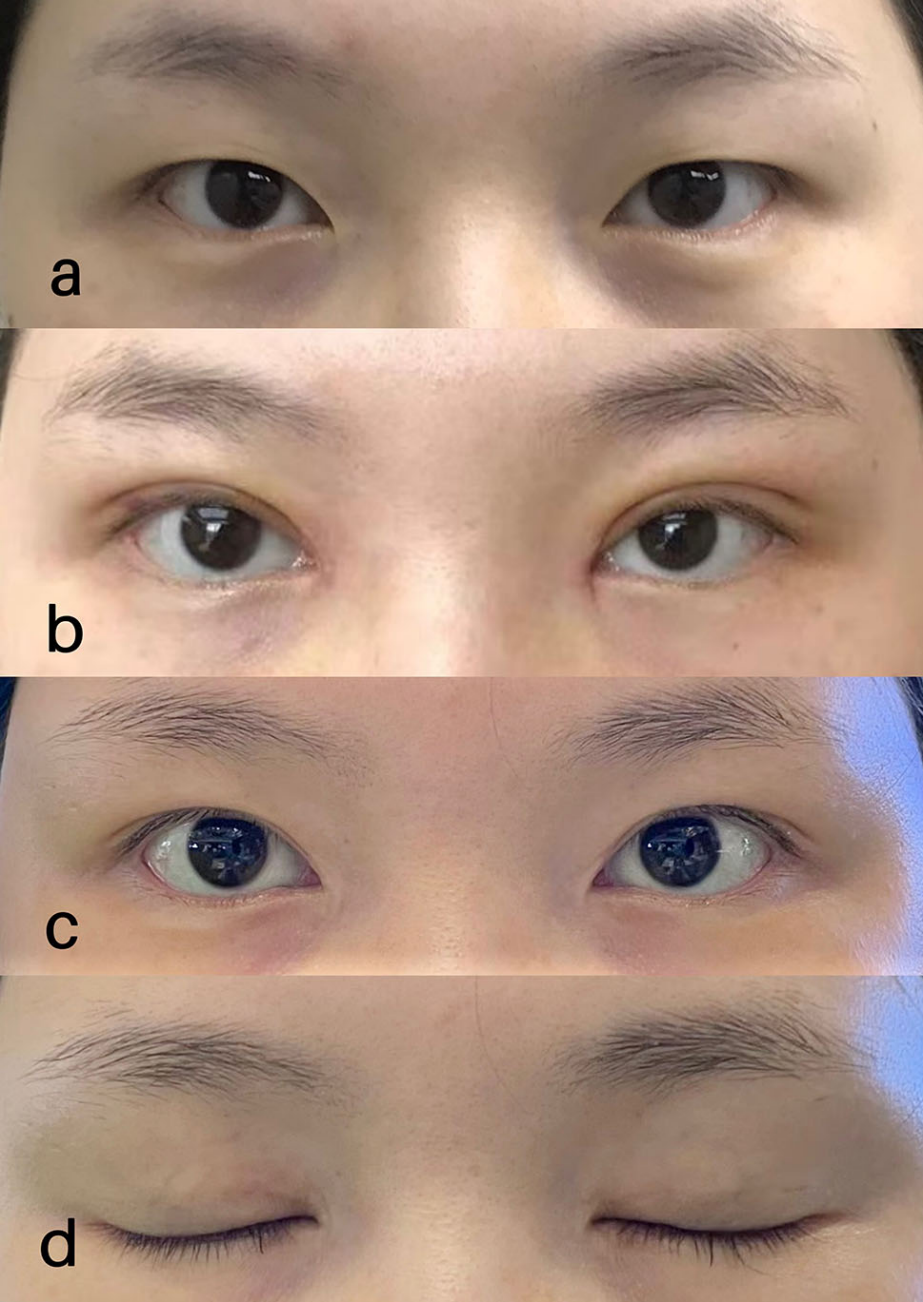
**a** Preoperative view. **b** Seven days after blepharoplasty. **c** Six months after blepharoplasty. **d** Closed eyes after six months

The patient (0.2%) had asymmetries in the palpebral height and loss of the crease. The majority of other indicators showed a good fold curve. These patients were satisfied with the revision effects, and no reoperation was needed. Typical cases with representative outcomes are shown in Figs. 4 and 5.

## Discussion

In East Asian eyelids, the tendon fibers form a weak attachment to the dermis at a lower level. This, together with the sagging of the orbital septum fat, results in a less-pronounced eyelid crease in East Asians than in other populations [2]. This has made double-eyelid blepharoplasty the most popular plastic surgery in Asia.

Due to the complications associated with traditional methods, numerous doctors prefer to preserve the orbicularis. In 1999, Park [8] proposed a surgical method for retaining the orbicularis by suturing the tissue with the levator aponeurosis. Because the pretarsal orbicularis was intact, the incision scar was not noticeable [7, 9, 11]. However, due to the cutting force and eye-opening movement after surgery, there were sometimes problems involving palpebral fold loss and asymmetry after the procedure [9, 12].

Many surgical teams have followed up on blepharoplasty research by improving the position of the sutures to prevent suture cutting and double-eyelid shedding [10, 11, 13, 14]. The essence of bionic blepharoplasty is to connect the orbicularis to the levator aponeurosis. Because power conduction is not directly associated with the tarsus, there is still a risk of the double-eyelid fold loosening and changing in width in the long term.

An orbicularis–levator–tarsus composite suture technique was then proposed [15]. In this method, the tarsus, levator aponeurosis, orbicularis, and orbital septum are sutured into a firm fixation. Sun et al. [16] wrote that neither the Park method nor composite suturing can prevent shifting and looseness of the fixed position with the movement and aging of the aponeurosis. In this work, we modified Park's technique. In our patients, the orbicularis was directly fixed to the meibomian to create a solid adhesion.

Our study aims to address these problems through innovation. We used selective opening instead of complete incision, which can reduce damage and injury to the orbicularis.

This procedure has the following advantages:

1. Forming a levator aponeurosis–tarsus–orbicularis–skin linkage mechanism. The underlying principle of levator aponeurosis surgery is the direct connection between the aponeurosis and the orbicularis. The levator aponeurosis directly tugs on the orbicularis when the eyes open, which increases the tension on the double-eyelid incision. Our study strengthens the fixation of the entire tarsus and the orbicularis. It forms a double-eyelid indirectly through the natural connection between the levator and the tarsus. This linkage mechanism is a multi-step movement, which can disperse the incision tension during the opening of the eyes such that the width of the double-eyelid does not vary with the strength of the levator. It also reduces the suture cutting caused by the change in tension as the eyes open and close [16].
2. Surface adhesion. The levator aponeurosis of a natural single eyelid is weaker and the pre-tarsal tissue is thicker than those of a natural double eyelid. In previously proposed levator aponeurosis surgical techniques, the fixation mechanism used is linear [10, 13], which significantly affects the stability. Adhesion is directly affected by the cutting force of the suture and the tension produced when the eyes open. It is part our surgical procedure to remove the pre-tarsal tissue, which plays a lubricating role between the tarsus and the orbicularis [15, 17]. Removing the pre-tarsal tissue causes scars to heal, which can foster adhesion between the orbicularis and the tarsus, also known as surface adhesion.
3. Selective opening of the orbicularis. We here adopted the concept of preserving orbicularis from Park’s technique [8]. However, unlike the complete incision of the orbicularis [11, 14, 18], our procedure removes the pre-tarsal tissue, and even the orbital septal fat, through two selective openings to maximize the continuity of the orbicularis and reduce unnecessary injury.
4. Tissue in situ suture. Unlike the suture of levator aponeurosis and orbicularis, our study adopts the concept of muscle and muscle closure, skin and skin closure and completely anatomically resets all levels to prevent scar depression.
5. Operability. Our study reduced the steps to find the levator aponeurosis. This reduces the intraoperative anatomical damage, reduces the difficulty of surgery, and reduces the operation time. This method shortens the learning curve for young surgeons.

## Conclusion

A modified double-eyelid blepharoplasty with the tarsus linkage mechanism was proposed in our study. It innovatively introduces the selective opening of the orbicularis to remove the pre-tarsal tissue and the herniated orbital fat without incising the orbicularis. Connecting the orbicularis and the tarsus in situ suture turns the lower flap of the eyelid into a composite unit. Compared to the orbicularis–aponeurosis connection, this connection provides a robust and stable eyelid adhesion.

This method is suitable for most primary eye cases, particularly in patients with lax upper lid skin and high levels of upper orbital fat. However, in clinical practice, due to the minimally invasive nature of the procedure, it is not possible to correct ptosis intraoperatively at this time. This issue warrants more in-depth study.

### Supplementary Information

Below is the link to the electronic supplementary material.

Supplementary file1 (WMV 260535 KB)

Supplementary file2 (WMV 5885 KB)
